# Lipids in *Aspergillus flavus*-maize interaction

**DOI:** 10.3389/fmicb.2014.00074

**Published:** 2014-02-27

**Authors:** Marzia Scarpari, Marta Punelli, Valeria Scala, Marco Zaccaria, Chiara Nobili, Matteo Ludovici, Emanuela Camera, Anna A. Fabbri, Massimo Reverberi, Corrado Fanelli

**Affiliations:** ^1^Dipartimento di Biologia Ambientale, Università Sapienza – RomaRoma, Italy; ^2^Unità Tecnica Sviluppo Sostenibile ed Innovazione del Sistema Agro-industriale, Laboratorio Innovazione Agroindustriale, ENEA C.R. CasacciaRoma, Italy; ^3^IFO-S.GallicanoRoma, Italy

**Keywords:** maize kernels, aflatoxins, lipoxygenase, lipidomic, reverse genetic

## Abstract

In some filamentous fungi, the pathways related to the oxidative stress and oxylipins production are involved both in the process of host-recognition and in the pathogenic phase. In fact, recent studies have shown that the production of oxylipins in filamentous fungi, yeasts and chromists is also related to the development of the organism itself and to mechanisms of communication with the host at the cellular level. The oxylipins, also produced by the host during defense reactions, are able to induce sporulation and to regulate the biosynthesis of mycotoxins in several pathogenic fungi. In *A. flavus*, the oxylipins play a crucial role as signals for regulating the biosynthesis of aflatoxins, the conidiogenesis and the formation of sclerotia. To investigate the involvement of an oxylipins based cross-talk into *Z. mays* and *A. flavus* interaction, we analyzed the oxylipins profile of the wild type strain and of three mutants of *A. flavus* that are deleted at the Af*lox1* gene level also during maize kernel invasion. A lipidomic approach has been addressed through the use of LC-ToF-MS, followed by a statistical analysis of the principal components (PCA). The results showed the existence of a difference between the oxylipins profile generated by the WT and the mutants onto challenged maize. In relation to this, aflatoxin synthesis which is largely hampered *in vitro*, is intriguingly restored. These results highlight the important role of maize oxylipin in driving secondary metabolism in *A. flavus*.

## Introduction

*Zea mays* is one of the most cultivated grain crop. The yield, quality and safety of its kernels are continuously challenged by pathogens. Amongst these, the mycotoxigenic fungi are actually the most health hazardous. *A. flavus* under suitable conditions infect maize kernels both during development in the field as well as during the storage (Sheidegger and Payne, 2003). In most cases *A. flavus* produces the harmful and carcinogenic aflatoxins, among which B1 is considered by IARC (International Agency for Research on Cancer) as belonging to group 1, i.e., carcinogenic to humans and animals. Seed composition, notably lipid composition, may affect susceptibility to fungal infection and mycotoxins production (Reddy et al., 1992; Dall'Asta et al., 2012). Unsaturated fatty acids (FAs) have been frequently described as modulators of plant resistance pathway upon pathogen attack, even though their profile is strongly influenced by the environmental conditions experienced by plants during the entire growing season (Kachroo and Kachroo, 2009; Dall'Asta et al., 2012). Polyunsaturated FAs, released from membranes by lipases in response to attacks by biotic agents, play a key role in plant-pathogen interaction either directly as free FAs or as precursors of oxylipins (Walley et al., 2013). As an example, linoleic acid levels contribute to the regulation of development, seed colonization and mycotoxin production in *Aspergillus* spp. (Calvo et al., 1999). FAs may also serve as precursors in oxylipins synthesis. These oxidized FA-derived compounds are involved in plant-fungi interaction. It is possible to suggest the presence of an “oxylipins signature profile” typical for every pathogen (Tsitsigiannis and Keller, 2007; Reverberi et al., 2010). Similarly, plants contain phyto-oxylipin pools conferring an oxylipin signature on a given organelle, tissue or plant (Blée, 2002; Camera et al., 2004). Lipoxygenases (LOX) are amongst the main oxylipin-producing enzymes in all organisms. LOX are widespread in animals, plants and fungi, and their products have a variety of biological functions, involved in important physiological processes, for instance, the events of infection (in plants) or inflammatory events (in animals) and the structural and metabolic changes in animal cells. LOX enzymes have been found in plants, animals, fungi, and in all organisms that possess the required substrates, i.e., PUFAs (Polyunsaturated Fatty Acids) (Feussner and Wasternack, 2002).

Lipoperoxidation seem to play an important role in the induction of the production of certain mycotoxins including aflatoxins. *In vitro*, synthetic inducers of lipid peroxidation, such as cumene hydroperoxide, stimulate the production of aflatoxins, when added to culture media inoculated with toxigenic strains of *A. flavus* and *A. parasiticus* (Passi et al., 1985). *In vivo*, the amount of toxin produced on sunflower seeds differs according to the age of seeds and the amount of peroxides found in the oil obtained from them; older will be the seeds, the greater the number of peroxides, and will be higher the amount of aflatoxin produced by these fungi (Passi et al., 1984). Several studies have shown how oxylipins in plants are capable of inhibiting the mycelial growth and germination of spores (Prost et al., 2005). The similar structure between the oxylipins of fungi and plants suggest the hypothesis that these molecules are very important for the host-pathogen communication.

According to Tsitsigiannis and Keller (2007), oxylipins are able to modulate the sporulation through specific transcription factors related to cleistothecia (NsdD) and conidiospores (BrlA) formation; they may control homeostasis through the lipogenic transcription factors SREBP -1 and SREBP -2 and induce the development of the fungus and the production of secondary metabolites, such as penicillin, through the pathway of G proteins involving protein kinase A (PkaA). Studies on *A. nidulans* and *A. fumigatus* has led to hypothesize that oxylipins are able to escape from the cell through specific transport proteins, act as autocrine or paracrine ligand with membrane receptors, GPCRs, both of the fungal cell itself than that of the host, and activate a downstream signaling cascade (Tsitsigiannis and Keller, 2007).

Linoleic acid host-derived oxylipins may drive aflatoxin synthesis, as widely demonstrated (Burow et al., 1997; Brodhagen et al., 2008; Reverberi et al., 2012). The oxylipin-driven actions are further complicated by the evidence that even fungal pathogens produce oxylipin during the interaction with the host. A recent evidence of the central role of oxylipins in the regulation in the biosynthesis of mycotoxins and fungal sporulation was obtained in *A. ochraceus* interacting with viable seeds of wheat (Reverberi et al., 2010). In this study, a mutant defective for a *lox*-like gene produces minimal amounts of 13S-HPODE and show a strong decrease in the production of ochratoxin A, a delayed formation of conidia and an increase in the production of sclerotia. Furthermore, seeds infected with this mutant are not able to accumulate normal levels of 9S-HPODE nor to induce the expression of the gene of defense PR1, suggesting that the fungal oxylipins may modulate the response of the host defense. These data demonstrate that, the plants oxylipins influence the processes of development of *Aspergillus* because of their similarity with those of fungi. It has been observed that the PSIB α oxylipins of *A. nidulans*, derived from linoleic acid, are very similar to those produced from fatty acids of seeds and that these, after infection, are able to regulate the development of the fungus mimicking and/or interfering with the signals that regulate the processes sporogenesis (Prost et al., 2005).

In this paper we demonstrate that combining previous microarray data (Reverberi et al., 2013) with reverse genetic approach and with a lipidomic procedure may provide evidence of the key role played by lipids and oxylipins in the interaction between *A. flavus* and maize kernels, notably in influencing aflatoxin biosynthesis.

## Materials and methods

### Fungal strains and culture conditions

*Aspergillus flavus* NRRL 3357, aflatoxin B_1_ producer and an argD/uracil double auxotroph mutant (AFC-1) (Zhu-Mei et al., 2007) were used in these studies. An Af*lox1*Δ transformant was generated from AFC-1 as described below. The fungal strains were maintained on Czapek Dox Agar (CDA) (Difco), amended with ZnSO_4_ (5 mg/L) and NaMoO_4_ (1 mg/L), for 7 days at 30°C.

### Fungal growth, conidiogenesis, and aflatoxin production

#### In vitro assays

*Fifty* ml of Potato Dextrose Broth (PDB) (Difco, USA) in 100 mL Erlenmeyer flasks were inoculated with the WT or the 3 strains of Af*lox1*Δ (1.1; 3.2; 5.1), using 0.1 mL of conidial suspension (~10^6^ conidia) for each flask (*n* = 3); the cultures were then incubated at 30°C for different time periods (0–14 days post inoculation; total *n* = 5 time intervals). *N* = 2 biological replica were carried out. At each point in time, fungal growth was determined by weighting the mycelium after filtration (Millipore filters, 0.45 μm) and drying it for 48 h at 80°C (d.w.). Conidia formation was determined at each time interval by washing the mycelia with a solution of triton 0.01% w/v, taking 0.5 ml of this solution and calculating conidia's number using a hemacytometer. The filtered mycelia were lyophilized and weighed for determining the amount of oxylipins and performing molecular analyses.

#### In vivo assays

Each stock of maize seeds (30 g) was superficially sterilized (2% v/v NaClO solution), rinsed threefold by sterilized distilled water, moistened up to 0.90 a_w_ and then inoculated with 250 μ L of *A. flavus* conidia (*n* = 3 Erlenmeyer flasks for each test) suspension (10^4^ conidia/mL) at 30°C. Each biological replica (*n* = 2) consisted in non-contaminated (control) and contaminated seeds of maize kernels, harvested at *n* = 5 different time intervals (from 0 up to 14 days post inoculation, dpi).

Aflatoxin production was analyzed from cultures of the WT and Af*lox1*Δ strains grown both in PDB and onto viable maize seeds following extraction with chloroform:methanol (2:1 v/v) procedure. The extracts were collected, the volume was reduced under a stream of nitrogen and the quantitative analyses were carried out by HPLC, as previously reported (Reverberi et al., 2008).

### Plasmids and transformation

To obtain the disruption cassette for the Af*lox1* gene,5′- and 3′- flanking ends of the gene have been amplified, inserting in the primers (see below) a complementary tail to the 5′-end (*primer* #3) and 3′ (*primer* #4) of the *argD* gene; *argD* either has been amplified by inserting into the primers tails complementary to the 3′-end of 5′-flanking region (*primer* #2) and to the *5′*-end of the 3′-flanking region (*primer* #5)

#1 (*for*) 5′-AAAGGCTGGCACGTAGAAGA-3′

#2 (*for*) 5′-CCTAGTCGAGACGGAAAACGTAATTGCGGAGCAAATCACA 3′

#3 (*rev*) 5′-TGTGATTTGCTCCGCAATTACGTTTTCCGTCTCGACTAGG-3′

#4 (*for*) 5′-GAATCCCTGCATCAGAGGAATCAATTCCATCATTCCACGA-3′

#5 (*rev*) 5′-TCGTGGAATGATGGAATTGATTCCTCTGATGCAGGGATTC-3′

#6 (*rev*) 5′-CTACTGTGGCCTTTCCCAAA-3′

DNA extracted (50 ng) from *A. flavus* NRRL 3357 was amplified in a thermal mastercycler gradient (Eppendorf, Germany) following amplification steps (95°C × 2 min; 95°C × 30 s, 55°C × 45 s, 72°C × 1 min × 35 times; 72°C × 8 min). 5′-flanking region (1386 bp) has been amplified by *primers* #1 and #3; *argD* gene fragment (2217 bp) by *primers* #2 e #5; 3′-flanking region (1321 bp) by *primers* #4 and #6. After the obtainment of the 3 fragments, a PCR with external primers, #1 and #6, was carried on for obtaining gene disruption cassette; a DNA *mix* composed by 104 ng of 5′ *-flank* + 168 ng of argD + 100 ng of 3′*-flank* was used as template for the amplification. Protoplast transformations of *A. flavus* were performed by the polyethyleneglycol method as described elsewhere (Woo et al., 1999).

### Selection of transformants

The selection of Af*lox1*Δ transformant strains was conducted at 30°C on CDA containing 30 mM uracyle; putative transformants were selected, transferred to fresh selective medium, and allowed to sporulate. To obtain homokaryons, single spores were isolated from each selected heterokaryotic transformant and transferred to fresh selective medium. This monoconidial transfer was conducted three times. Finally, 20 monoconidial progenies were selected and further sub-cultured to determine the occurrence of abortive transformants. The stability of these transformants was also tested by two additional single-spore transfers on non-selective medium and then again on selective medium, and by several mycelial transfers on selective plates.

### Southern blot hybridization

For Southern blot analysis, 10 μ g of genomic DNA from *A. flavus* NRRL 3357 and Af*lox1*Δ was completely restricted with *Eco*RI (10 U) at 37°C for 4 h in the manufacturer's buffer at the recommended concentrations (Fermentas, Germany). *EcoR*I-digested DNA fragments were separated by electrophoresis for 3 h and 30 min at 40 V on 0.8% w/v agarose gel in TAE buffer. DIG-labeled *Hin*dIII cut lambda (λ) (Roche, Swiss) was used as MW standard. Fluorescent DNA probes were prepared according to the PCR DIG-labeling mix method (Roche, Swiss). The membranes were pre-hybridized according to the instructions of the manufacturer of the DIG- detection kit, at 64°C in DIG-easy buffer (Roche, Swiss); they were then hybridized for 12–16 h in the same buffer containing 250 ng of freshly denatured digoxigenin *argD* probe at 65°C.

### RT-PCR analyses

Total RNA from 100 mg of freeze-dried mycelia was extracted using the Tri-Reagent protocol (Sigma-Aldrich, USA) and was quantified by spectrophotometry, determining the optical density at 260 nm. RNA was treated with RNAse-free DNAse I and then re-suspended in 20 μL of DEPC-treated water. RNA was extracted at different points in time (from 24 to 168 hpi; 3 tubes for each point in time) from *A. flavus* WT and Af*lox1*Δ CDB cultures and was used to develop *lox1* SYBR green RT-PCR assay, as previously reported (Reverberi et al., 2010). Gene expression in the WT strain and the Af*lox1*Δ transformants were also measured by comparing mRNA levels in the different time intervals with their own basal expressions at the baseline, i.e., after conidia germination (time 0). *A. flavus* β-tubulin RNA was used as the housekeeping gene to normalize the differences in total RNA target input and quality and in RT efficiency, using specific primers as Afβ tub (Afβ tub_for GGAAGTCAGAAGCAGCCATC; Afβ tub_rev GTGACCACCTGTCTCCGTTT).

### Lipoxygenase assay

The LOX activity in the WT strain and in the Af*lox1*Δ transformants grown in PDB medium was assayed 0 to 14 days after inoculation using a Beckman DU 530 spectrophotometer by following up the formation of conjugate dienes at 234 nm as previously reported (Reverberi et al., 2008). In order to exclude the possible interference of laccase activity, KCN 1 mM was added to the reaction mixture before the spectrophotometric assay (Gülçin et al., 2005).

### Lipidomic untargeted analysis

Free, conjugated and modified fatty acids were extracted as described by Stumpe et al. (2005) with slight modifications. An amount of maize kernels (100 g) and/or mycelia (20 mg) were lyophilized and ground in liquid nitrogen. An aliquot of 20 mg was collected in a clean tube and added of 1 mL of the extraction medium (hexane: 2-propanol 3:2 v/v, containing 0.0025% butylated hydroxytoluene w/v) and 5 μ L heptadecanoic acid standard solution 2 mg/ml in EtOH added as the internal standard reference as reported in Scala et al. (2013). Separation and accurate mass measurements of lipid compounds was performed with a 1200 series rapid resolution HPLC coupled with a G6220A series time of flight mass spectrometer (ToF-MS, Agilent Technologies, CA, USA) equipped with an electrospray (ESI) interface operating in the negative ion mode. LC/MS-ESI ToF data were acquired and deconvoluted into individual chemical peaks using the Mass Hunter™ acquisition software. Untargeted and semi-targeted mining of the HPLC/MS-ESI ToF data were performed with the molecular feature extraction (MFE) algorithm in the Mass Hunter™ software. All the analyses were performed as previously described in Scala et al. (2013).

### Statistics

All the experiments were carried out in three replicates of two biological replica. The values presented in figures and tables are the mean ± SE of 6 different results. The mean values were compared by using the Mann-Whitney test; *p*-values above 0.05 were considered not significant. Analysis of variance (ANOVA) was applied in the comparison of the treatments, and significance of differences were tested at 95% confidence by Fisher's LSD test which is a least significant difference (LSD) method consisting in a two-step testing procedure for pair wise comparisons of several treatment groups. Calculations were performed using XLSTAT Addinsoft software [45]. Concerning the lipidomic approach, the MFE algorithm was used to extract individual molecular species by their accurate mass detected with the ToF MS. The detected species were characterized by accurate mass, isotopic pattern and absolute abundance and lists of molecular features were generated from each analyzed sample and converted into compound exchange files (CEF), which were then processed with Mass Profiler Professional (MPP) 12 (see below). Molecular features detected in the HPLC-MS system were aligned by their retention time (RT) in the chromatographic runs, and accurate mass axis in order to compare their expression across the different maize hybrids in different growth stages. Compounds detected in the different samples and presenting consistent RT (shift below 6 % of the RT) and accurate mass (mass error below 6 ppm) were assigned as the same molecular species. Relative abundance of individual features was obtained by normalizing their peak area by the area of the ISTD. Among all the metabolites, only those features consistently detected throughout all the analyzed maize samples were selected for further statistical analysis. ANOVA with Tukey's *post-hoc* test was performed on the entities detected with 100% frequency in the different samples. Fold changes of filtered entities were compared between WT, mutant strains and maize alone and significance was determined by Student's *T*-test. Differential expression was evaluated at each sampling time, including harvest, and visualized by Volcano plots. Changes higher than 2-folds, with *p* < 0.05 after the Benjamini-Hochberg correction, were considered as significant. Principal components analysis (PCA) was then performed on entities filtered following grouping of samples according fumonisins amounts. Compound identification and annotation was performed using the METLIN Personal Metabolite Database by means of the ID browser tool, and the Molecular Formula Generator algorithm. The LIPIDMAPS (http://www.lipidmaps.org/) database was used to infer compound identity. The annotation of free fatty acids (Fas) reported the number of carbon atoms and of double bonds. Other lipids were annotated consistently with the names reported on LIPIDMAPS.

## Results

### A*flox1* deletion: molecular and physiological characterization of deleted strains

**Figure 1:**
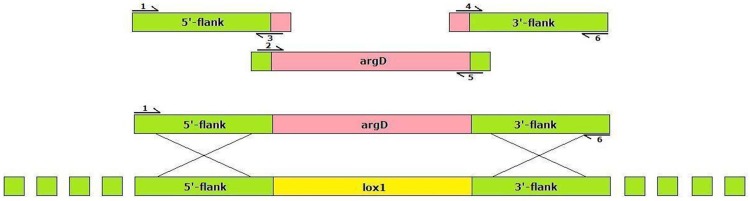
**Af*lox1* disruption cassette**.

Microarray analysis performed in previous study (Reverberi et al., 2013), indicated that a *lox* gene (corresponding to the affy probe 2911_m00089), coding for a Mn-dependent lipoxygenase (BLASTX result), was up-regulated in *A. flavus* during the pathogenic exploitation of maize kernels. To uncover the relation of oxylipin production in the fungus and in the maize kernels with aflatoxin synthesis Af*lox1*Δ strains have been generated by inserting a deletion cassette containing *argD* as selectable marker (AFC-1 strain is auxotroph for arginine—see Methods section) (Figure 1) and used for maize kernel infection.

**Figure 2:**
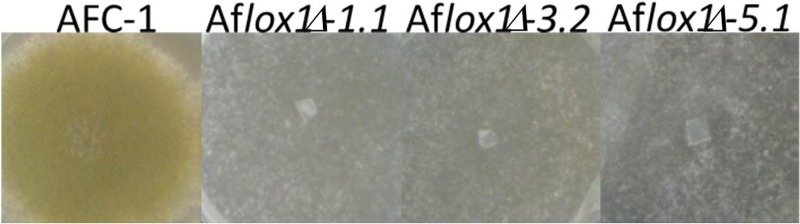
**Phenotypic comparison among Af*lox1*Δ strains with the native AFC-1 strain**.

Transformants selected as indicated in the Methods section, showed striking differences with the native strain AFC-1. These differences concern the growth (30–52% slower both in rich -PDB—and minimal—CD- media) and conidiogenesis (−90% compared to AFC-1) (Figure 2). This latter was completely recovered when Af*lox1*Δ strains were inoculated onto viable maize kernels (data not shown).

**Figure 3:**
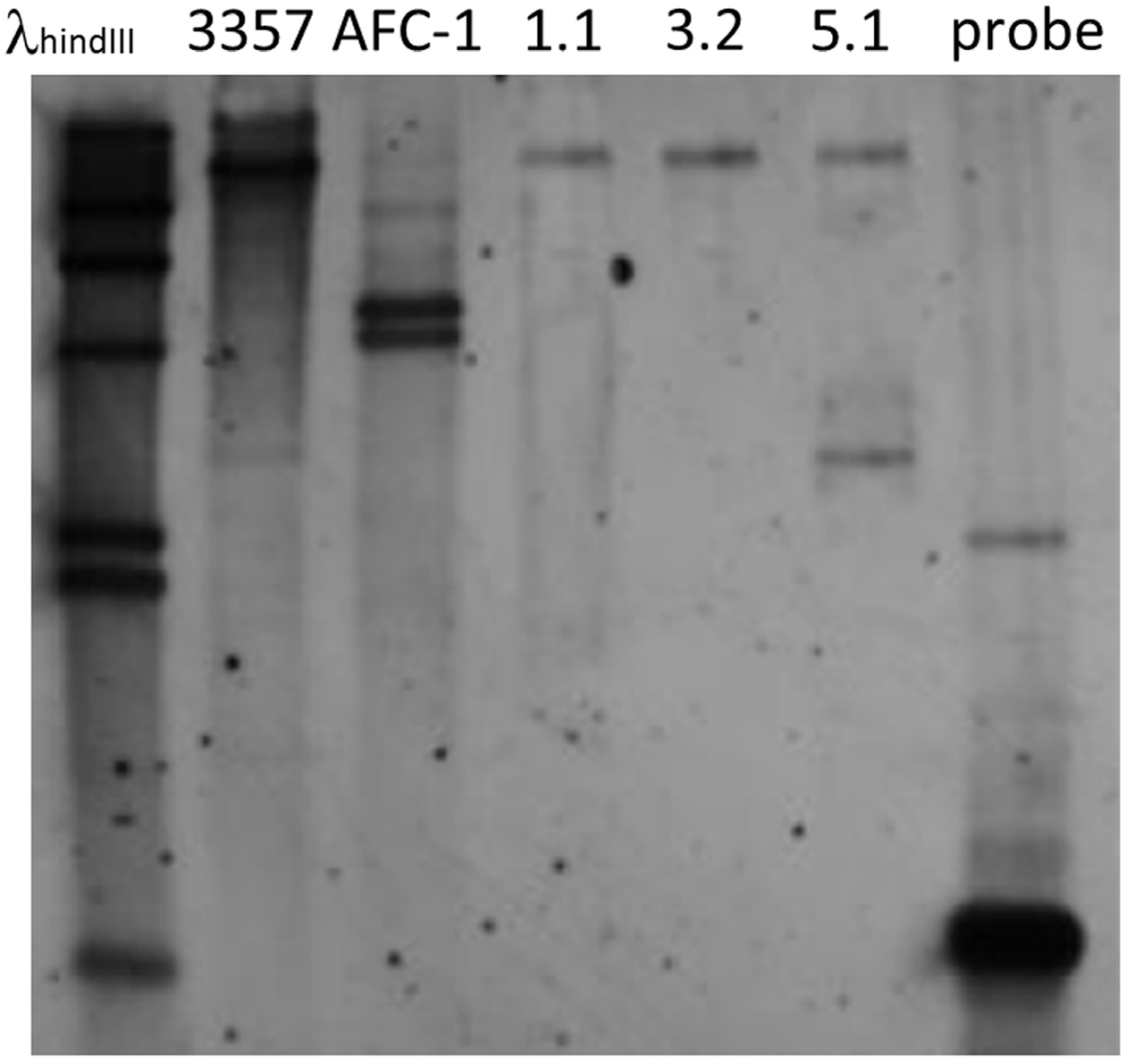
**Gel blot analysis of Af*lox1* gene replacement mutants.** Genomic DNA was isolated from the WT strain, the arginine auxotroph AFC-1 strain and gene replacement transformants (Af*lox1*Δ). The blot was hybridized at 65°C with a 0.65-Kb *argD* DIG-labeled probe. Lane 1 DIG-labeled λ_hindIII_ (Roche) used as molecular weight marker; lane 2 *EcoR*I-restricted genomic DNA of *A. flavus* WT strain; lane 3 *EcoR*I-restricted genomic DNA of *A. flavus* AFC-1; lane 4–6 *EcoR*I-restricted genomic DNA of Af*lox1*Δ strains (clone number 3 out of a set of 20 transformants screened); lane 7 *argD* 0.65 Kb PCR fragment.

To confirm and quantify deletion cassette insertion into Af*lox1*Δ strains a southern blot analysis has been performed by using *EcoR*I-digested genomic DNA of the putative Af*lox1* deleted mutants, AFC-1 and the WT with which AFC-1 was previously originated (i.e., *A. flavus* NRRL3357). Hybridization was performed by using an *argD* probe. Hybridization pattern confirm a single insertion of the deletion cassette into Af*lox1*Δ 1.1 and 3.2 strains whereas, a putative double insertion event can be suggested for Af*lox1*Δ 5.1 strain (Figure 3).

**Figure 4:**
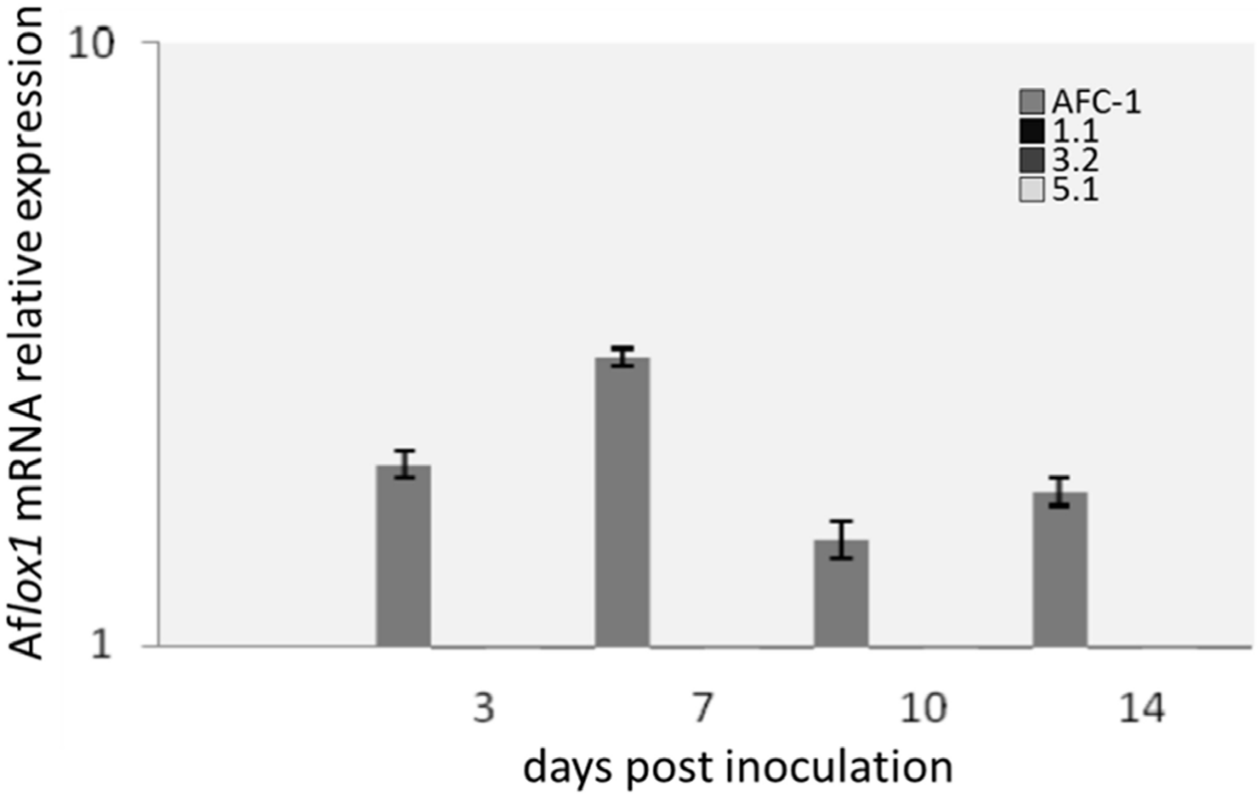
**Af*lox1* mRNA relative expression normalized to Af*lox1* expression at inoculation time 0 in *A. flavus* AFC-1 and Af*lox1* deleted strains (1.1; 3.2; 5.1) in PDB at 30°C at different days after inoculation (3–14 dpi).** Results are the mean (± SE) of a total of six replications deriving from two independent experiments.

Af*lox1* mRNA expression was analyzed into AFC-1 and in the 3 mutant strains selected for further analysis. All the fungal strains were grown in PDB and harvested at different time post inoculation (0–14 dpi). Results shown how *lox1* mRNA expression is under the detection limit into the Af*lox1*Δ strains also compared to gene expression into AFC-1 strain (Figure 4).

**Figure 5:**
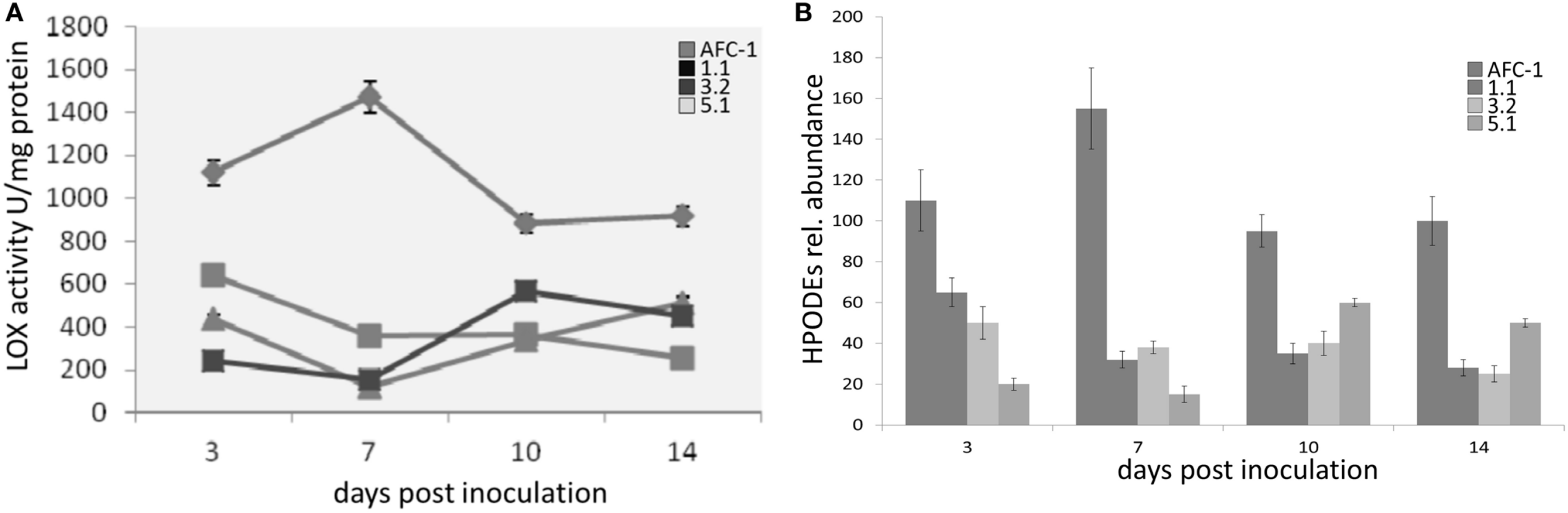
**(A)** LOX activity (U/mg protein) measured as diene conjugates formation at 234 mn in AFC-1 and Af*lox1* deleted strains in PDB at 30°C at different days after inoculation (3–14 dpi). **(B)** HPODE synthesis (expressed as relative abundance) in AFC-1 and Af*lox1* deleted strains in PDB grown at 30°C at different days after inoculation (3–14 dpi). Results are the mean (±SE) of a total of six replications deriving from two independent experiments.

Since *lox1* should be present in multiple copies in *A. flavus* as well as in other toxigenic fungi (Reverberi et al., 2010) we quantify the amount of LOX activity, by quantifying diene conjugates at λ_234_, into AFC-1 and Af*lox1*Δ mutant strains. It emerges that even if severely unpaired, a residual LOX activity is still present into deleted strains even if its amount is significantly (*P* < 0.001) to AFC-1 (Figure 5A). LC-TOF analysis of lipids extracted indicated that the synthesis of HPODEs (the main LOX-related oxylipins—Feussner and Wasternack, 2002) is consistently hampered in all Af*lox1*Δ mutant strains compared to AFC-1 strain (Figure 5B).

**Figure 6:**
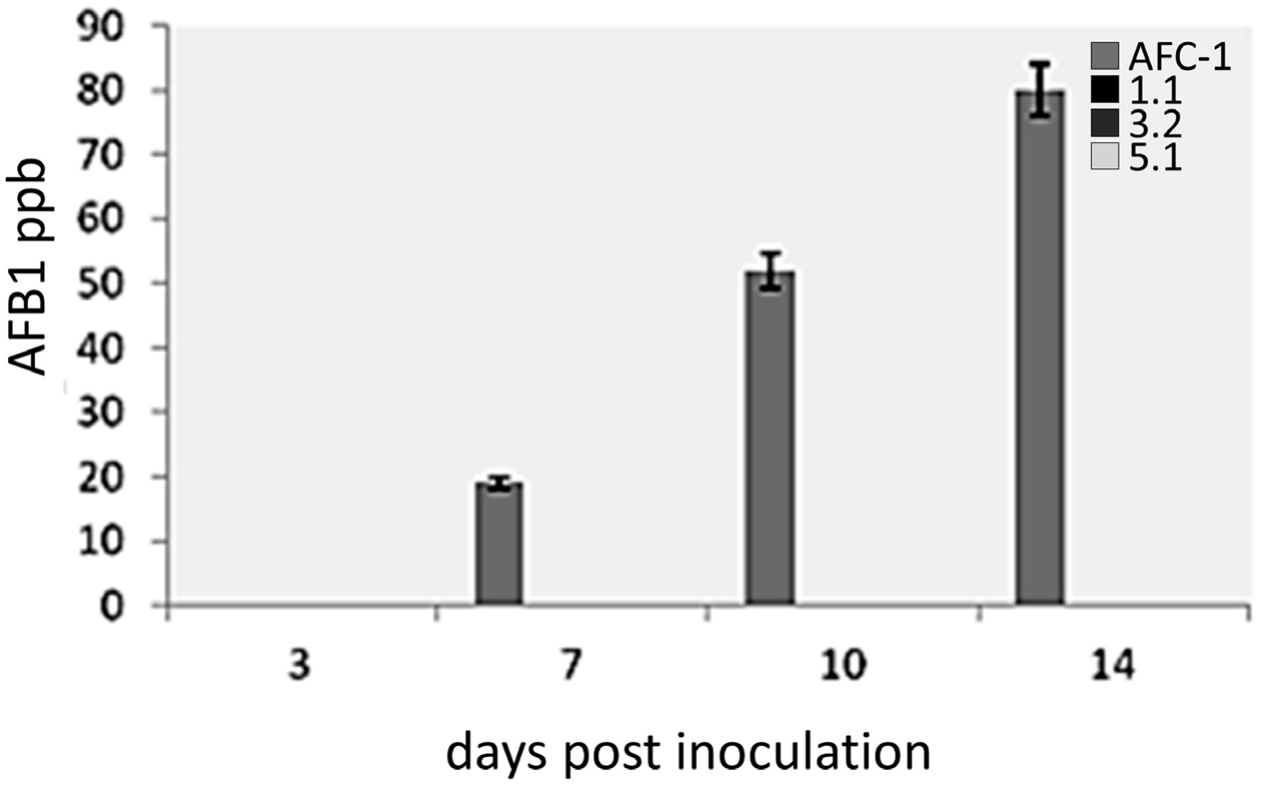
**Aflatoxin (AFB1) production measured by HPLC in culture filtrate (ppb), in the AFC-1 and Af*lox1*Δ strains grown in PDB at different time intervals of incubation (0–14 dpi) at 30°C.** Results are the mean (±SE) of a total of six replications deriving from two independent experiments.

Aflatoxin synthesis has been monitored at different time after inoculation (0-14 dpi) in PDB medium of the 4 fungal strains (AFC-1, Afl*ox1*Δ_1.1, Afl*ox1*Δ_3.2, Afl*ox1*Δ_5.1). It emerged that Af*lox1* deleted strains are unable to synthesize AFB1 in this culture conditions whereas AFC-1 has an AFB1 production similar to the native wild type NRRL 3357 (102 ± 15.2 ppb at 14 dpi) (Figure 6).

### Maize kernels inoculation with Af*lox1* deleted strains

**Figure 7:**
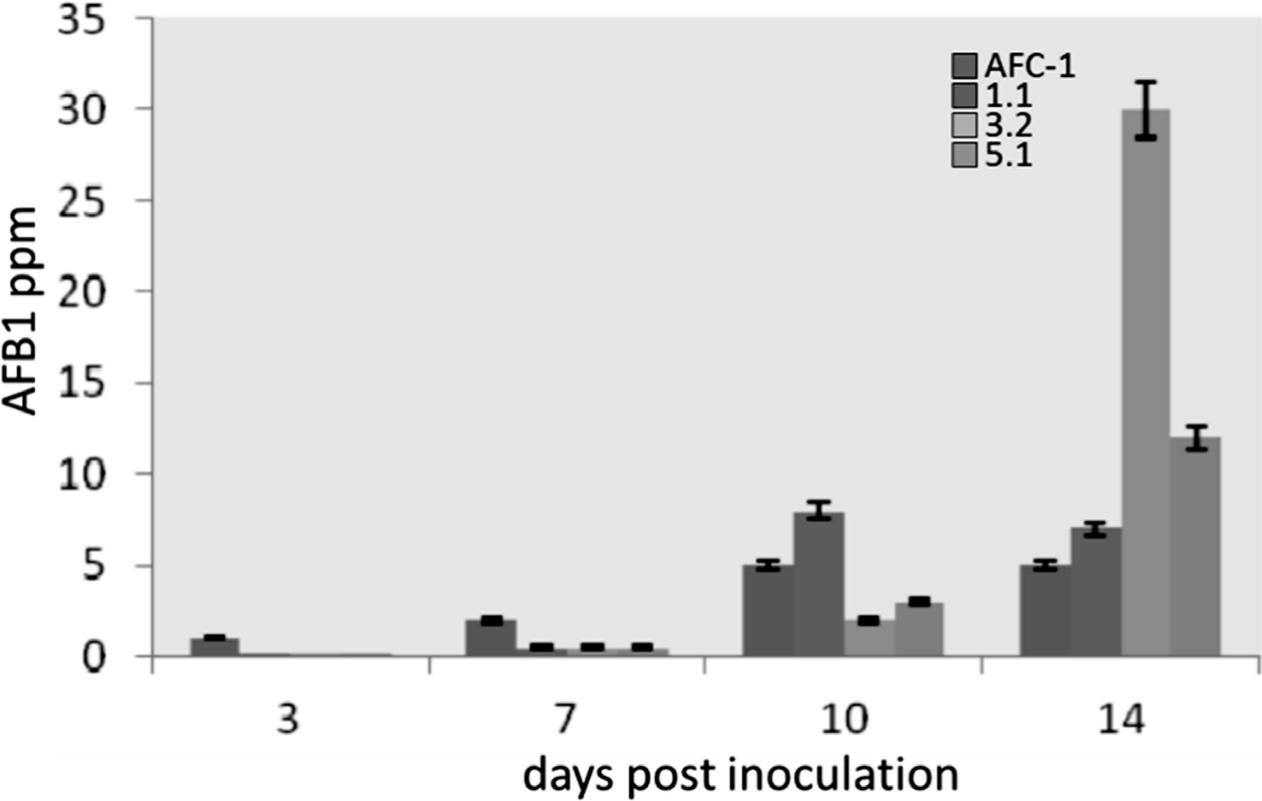
**Aflatoxins biosynthesis measured by HPLC (ppm) in viable seeds of maize by AFC-1, Af*lox1*Δ_1.1, Af*lox1*Δ_3.2, and Af*lox1*Δ_5.1 strains incubated at 30°C for different days post inoculation (3–14 dpi).** Results are the mean (± SE) of a total of six replications deriving from two independent experiments.

To study the behavior of Af*lox1*Δ mutant strains *in vivo* compared to the AFC-1 strain, an inoculum was performed directly on viable corn seeds in environmental conditions that mimicked the natural condition of pathogenesis. Since the mutants do not produce conidia, *in vitro*, the inoculation of was performed by a small portion (200 mg) of fresh mycelium into 10 g of viable seeds of maize, to make the test more homogeneous as possible, even the mycelium of the AFC-1 strain was inoculated in the same way, i.e., by inoculating the same portion of mycelium that had not differentiated conidia into maize seeds. The three mutant strains, already at early time of analysis (3 dpi), intriguingly recover the ability to produce conidia although the overall growth appeared anyway to be slower compared to the AFC-1 strain (data not shown). Aflatoxins biosynthesis was then evaluated in times of analysis similar to the *in vitro* test (3, 7, 10, and 14 dpi). The results of this analysis have highlighted the recovery, and in some cases the stimulation (in strain Af*lox1*Δ_3.2), of the capacity for synthesizing aflatoxin B1 in the three Af*lox1* deleted strains examined (Figure 7).

### Oxylipin profile into maize kernels challenged with *A. flavus*

**Figure 8:**
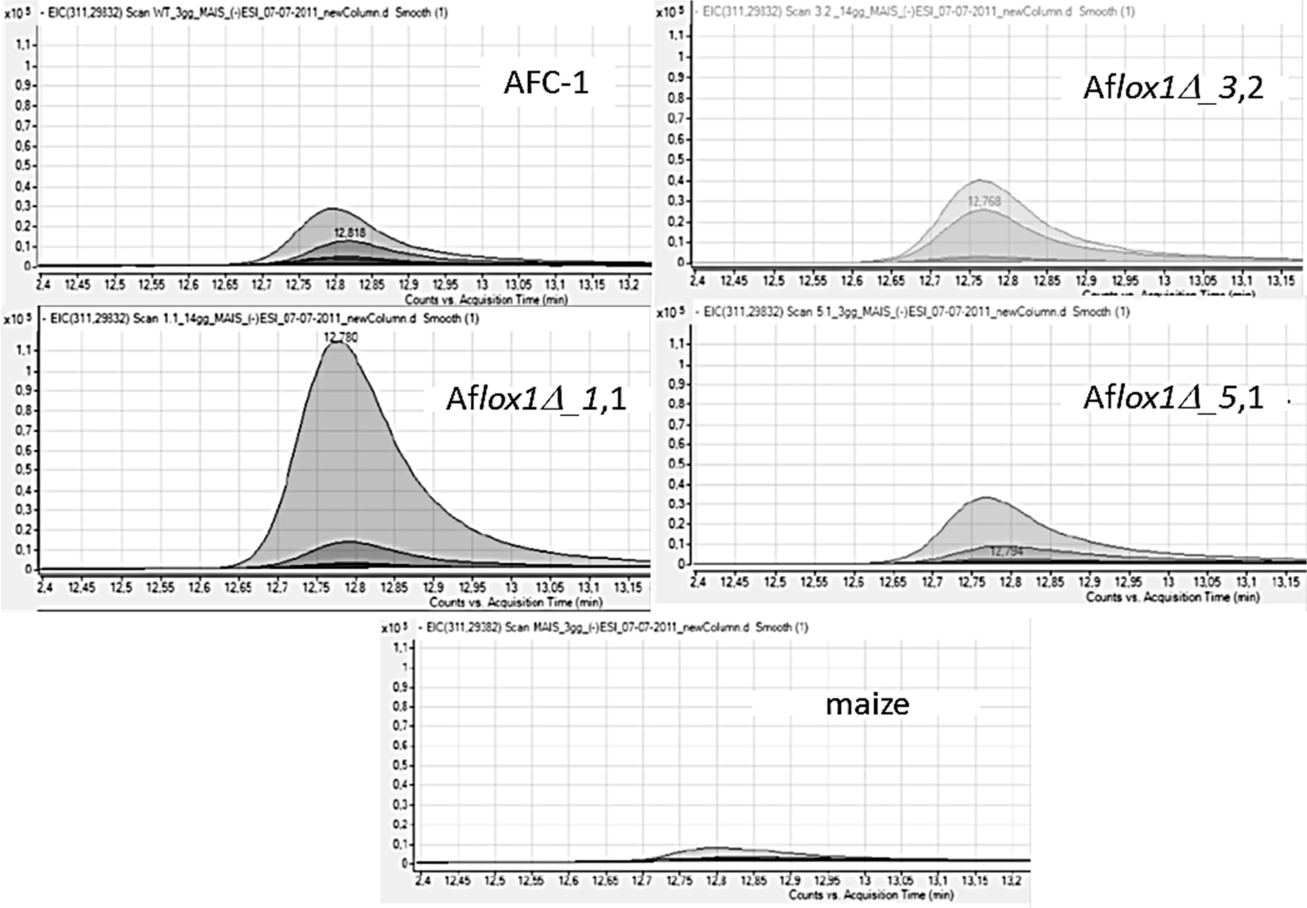
**Chromatogram (of the lipid compound at 311.2983 m/z corresponding to HPODE) in overlap mode deriving by LC-TOF analysis of lipid extract of maize kernels non-inoculated or inoculated with AFC-1 and the 3 deleted Af*lox1* strains (1.1/3.2/5.1) at 3, 7 and 14 dpi**.

Lipidome analysis and the characterization of specific oxylipin has been performed into maize kernels challenged with the 4 strains—1 native and 3 Af*lox1* deleted strains—of *A. flavus* (Figure S1). Through *Mass Hunter* software (Agilent tech, USA) it has been possible to pinpoint 5 oxylipins amongst the *plethora* of lipid compounds highlighted by lipidomic approach (Figures S2, S3). Notably, HPODEs, as described elsewhere (Burow et al., 1997), and di-HODEs result produced at a different amount by pathogen- challenged and unchallenged maize kernels. By simply comparing the chromatograms it is possible to highlight, concerning HPODEs, striking difference among the different samples, with particular regard to HPODEs produced by maize kernels inoculated with AFC-1 compared to those produced when the seeds were challenged with the 3 Af*lox1* deleted strains. Furthermore, the same analysis was carried out on maize seeds un-inoculated in the same experimental conditions. The HPODE profile of the not inoculated seeds is considerably lower compared to preceding, to indicate that the presence of the fungus can effectively elicit in somehow the production of oxylipin in maize too (Figure 8).

**Figure 9:**
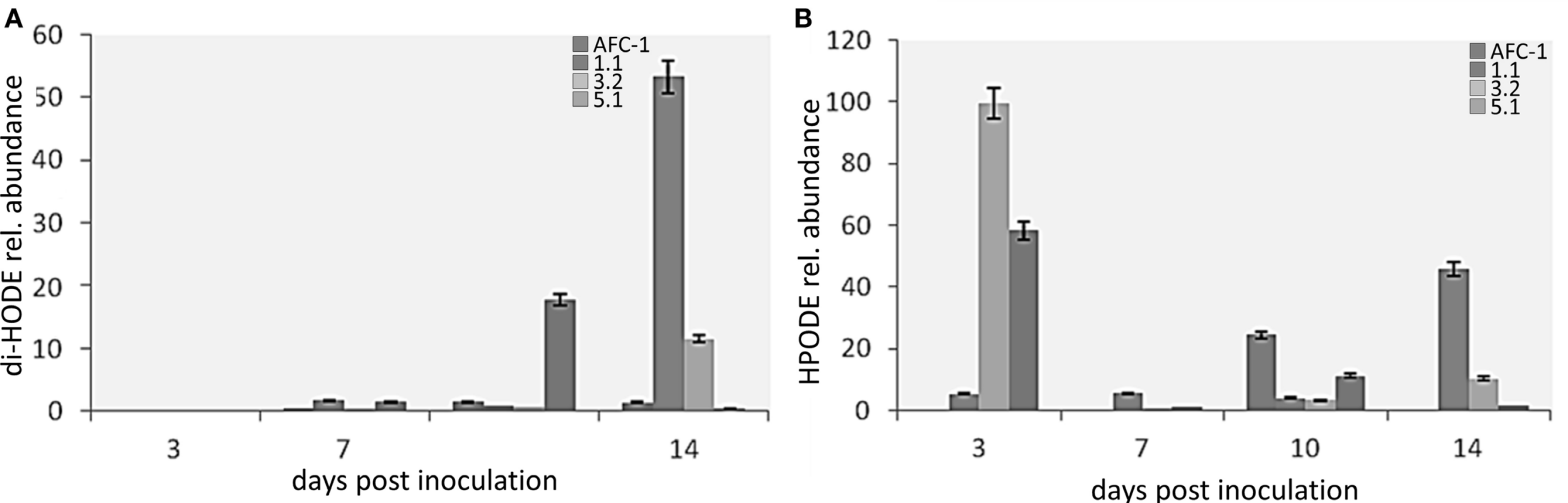
**Results in the production of (A) di-HODE and (B) HPODE in maize inoculated with the mutant strains or with AFC-1, 3, 7, 10, and 14 days post inoculation, compared to non-inoculated maize.** The results represent the average of six repetitions arising from two different experiments ± SE.

At this point, we evaluated the level of production of di-HODE and HPODE in inoculated relative to un-inoculated maize kernels. In both cases oxylipin production in the seeds infected by mutant strains results higher than that in the seeds infected with the WT (Figures 9A,B).

## Discussion

One of the principal concern related to the production of cereal seed is the contamination with mycotoxins produced by *Aspergillus* spp. and *Fusarium* spp. In particular, Aspergilli are commonly associated with crops such as maize, wheat, cotton, peanut, characterized by seeds rich in lipids, suggesting an important role played by the host lipids in influencing the ability of the pathogen to synthesize mycotoxins.

The role of lipids in the recognition and plant-pathogen communication has recently been revised and re-evaluated. Lipids and oxylipins currently represent one of the most effective control signals for morphogenesis, development and virulence of pathogens (Gao and Kolomiets, 2009; Christensen and Kolomiets, 2010; Reverberi et al., 2010).

Several studies have shown a direct correlation between the lipoperoxidative processes that occur in the seeds of various plant species (maize, sunflower) and the production of aflatoxins by *A. flavus* and *A. parasiticus* (Fabbri et al., 1983; Burow et al., 1997; Gao et al., 2009). In particular, experiments on maize have shown that the processes of lipoperoxidation in the seeds induce an alteration of the balance between oxidants and antioxidants, in favor of oxidants accumulation into the fungal cell thus stimulating biosynthesis of mycotoxins in *A. parasiticus* (Reverberi et al., 2007). The production of the toxins could be interpreted as the result of a fungal cell response to an incomplete scavenging of reactive oxygen species at the intracellular level (Reverberi et al., 2008; Hong et al., 2013).

Several studies showed that plants oxylipins are able of inhibiting the mycelial growth and germination of fungal spores (Prost et al., 2005). The similar structure found between fungal oxylipins and those of plants has led to the hypothesis that the latter can “mimic” the corresponding oxylipins acting directly on the physiology of the fungal pathogen try to alter its pathogenic behavior. Indeed, *in vitro* experiments have shown that linoleic acid and two of its oxidation products, namely 9S- and 13S-HPODE, play a significant role in differentiation processes in *A. nidulans*, in *A. flavus* and *A. parasiticus* (Burow et al., 1997; Gardner, 1998; Calvo et al., 1999; Gao et al., 2009).

Af*lox1* gene, Mn-lipoxygenase (LOX) encoding, knock-out mutants showed a significant reduction of mycelial growth and a complete inability to differentiate conidia and produce aflatoxins, if grown *in vitro* conditions. As shown by recent studies (Tsitsigiannis and Keller, 2007) in *A. nidulans* and *A. fumigatus*, the change in lipid metabolism has a strong influence on the production of sexual spores and the ability to produce toxins (Tsitsigiannis and Keller, 2006). The data obtained herein are therefore in line with the others reported in the literature. Intriguingly, inoculating Af*lox1*Δ mutants on viable and germinating maize seeds, it was possible to highlight the almost total recovery of conidiogenesis and production of aflatoxins. Oxylipins released from the seed, probably simulating/substituting fungal ones, induce the activation of secondary metabolism and changes in morphogenesis, as also suggested in Brodhagen et al. (2008). In these studies maize 9-LOX (Zm*LOX3*) was cloned in a mutant of *A. nidulans* unable to produce conidia. The oxylipins produced by the Zm*LOX3* gene are able to induce again the conidiogenesis, suggesting a strong correlation between host-oxylipins and production of conidia and providing a clear proof of the mutual cross-talk between plants and fungi mediated by oxylipins.

The LC-TOF and chemometric based lipidomic analysis of the maize kernels infected with the mutants and the wild-type strain of *A. flavus* showed a complete modification of the lipid and oxylipin profile. A higher production of HPODE and di-HODE in maize seeds inoculated with Af*lox1*Δ strains compared to those inoculated with the wild-type strain may be evidenced. The comparison with the production of the same oxylipins in non-inoculated maize seeds makes it possible to demonstrate not only that production is higher in seeds inoculated with the mutants, but also that the production of these particular oxylipins, is greatly increased if the host is interacting with the pathogen. To confirm this, it was also evaluated the production of HPODE and di-HODE in mycelia of the strains grew on media containing maize seeds (data not shown). The production was significantly reduced, showing that synthesis of these oxylipins is specifically charged to the seed. Recent studies have, in fact, show that LOX host genes expression is driven by the pathogen in *Aspergillus* infections leading to changes in the profile of the plant oxylipins (Tsitsigiannis and Keller, 2007). These results suggest that some derivatives of 9 -LOX may act as signal molecules for the production of mycotoxins and conidia. Experiments based on a knock-out mutant of the 9 -LOX gene of maize (Zm*LOX*3) confirm this hypothesis, showing that the lack of derivatives in the seed of the 9-LOX affect the pathogenesis and production of conidia and mycotoxins (Gao et al., 2007) and therefore, the importance of the role played by the host oxylipins for its exploitation by some pathogenic fungi. It can be suggested that the presence of the fungus (PAMP?) elicits the production of such oxylipins in the plant, which, in turn may influence the development of the fungus itself by stimulating certain physiological processes such as the production of conidia and the biosynthesis of toxin. The way in which these process aid pathogenesis is not yet elucidated.

### Conflict of interest statement

The authors declare that the research was conducted in the absence of any commercial or financial relationships that could be construed as a potential conflict of interest.

## Supplementary material

Figure S1 **Representative Total ion chromatogram of lipid compounds of AFC-1 grown on maize kernels after 7 dpi.** The different lipid compounds were sorted for exact mass and RT. A total of 1217 different lipid compounds were separated.

Figure S2 **PCA score plot of data generated by HPLC-ESI/TOF-MS analysis of of lipid extract of maize kernels non-inoculated or inoculated with AFC-1 (WT) and the 3 deleted Af*lox1* strains (1.1/3.2/5.1) at 3, 7, 10 and 14 dpi.** The results of the PCA analysis referred to three separate experiments performed in duplicate.

Figure S3 **Eulero–Venn's diagram showing compounds differences present in maize kernels inoculated with AFC-1 (WT) or the 3 deleted Af*lox1* strains.** The labels of each circle report the number of compounds selected on the basis of significant difference and 100% frequency in at least one group.
