# Supplementary figures and images for: Lipids in Aspergillus flavus-maize interaction
Source: Front Microbiol. 2014 Feb 27;5:74. doi: 10.3389/fmicb.2014.00074 (PMC3936598; doi:10.3389/fmicb.2014.00074)

## Slide 1
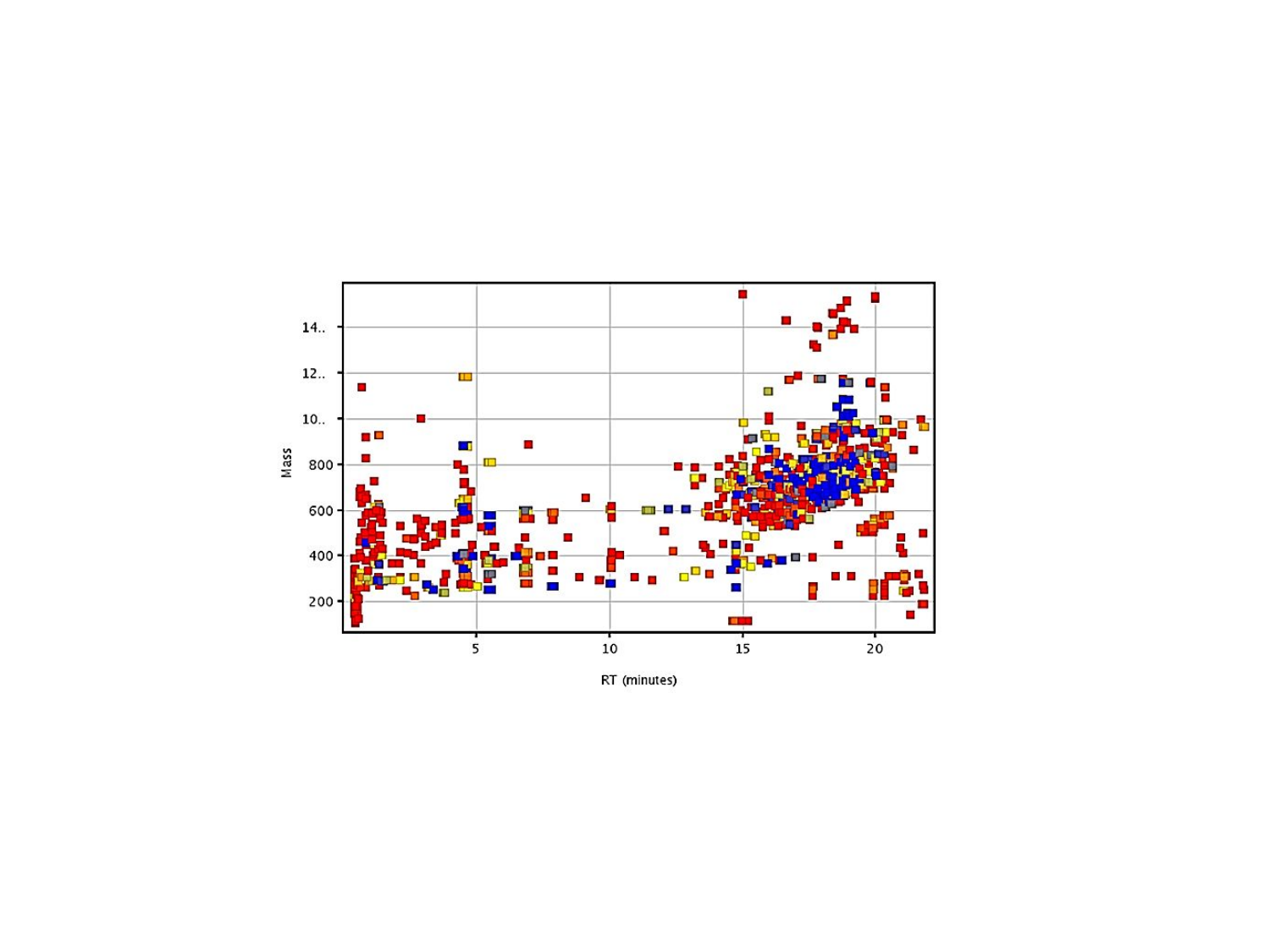

Supplement: Figure S1 — Representative Total ion chromatogram of lipid compounds of AFC-1 grown on maize kernels after 7 dpi. The different lipid compounds were sorted for exact mass and RT. A total of 1217 different lipid compounds were separated. [file Presentation1.PPTX]

## Slide 1
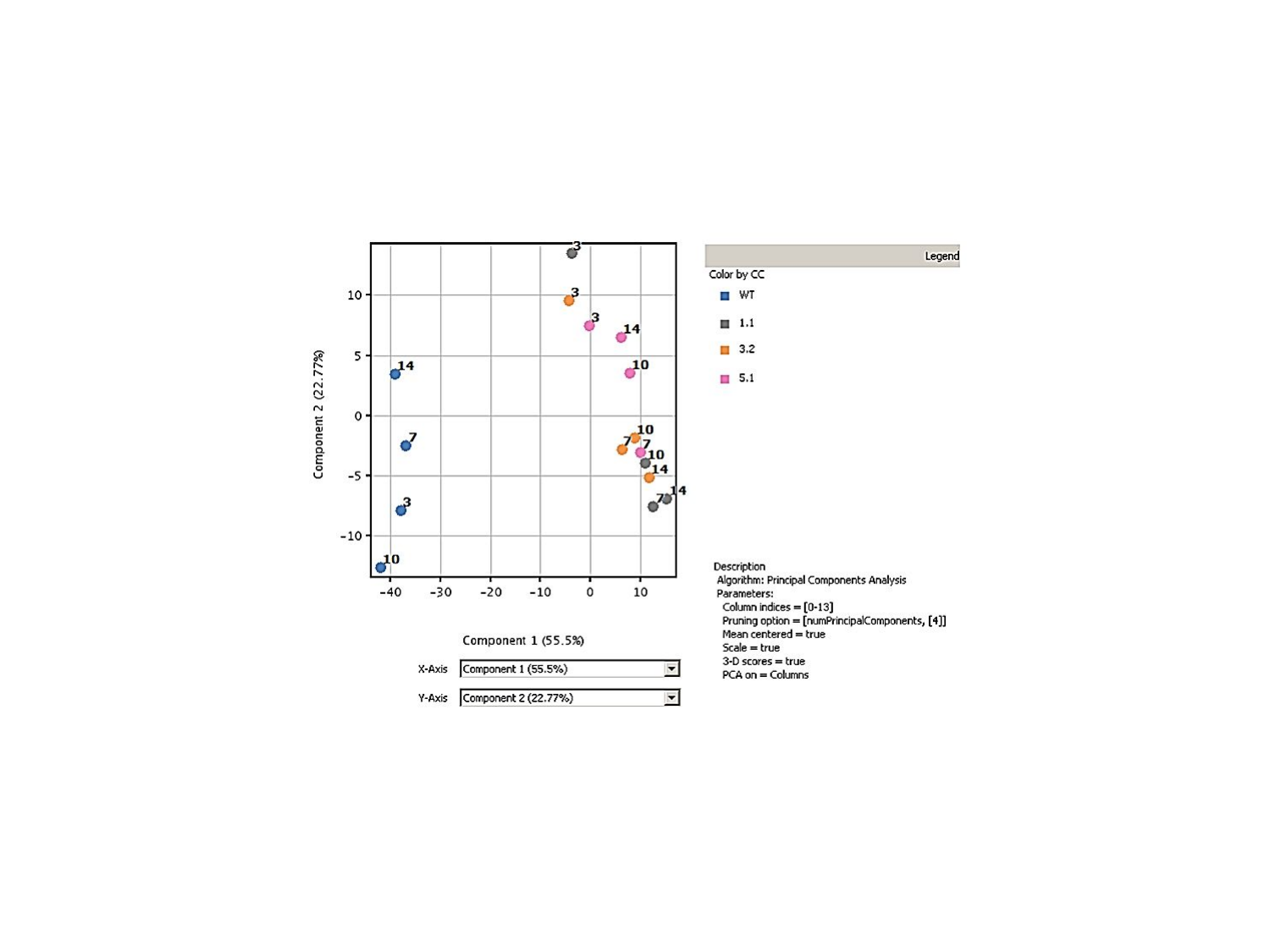

Supplement: Figure S2 — PCA score plot of data generated by HPLC-ESI/TOF-MS analysis of of lipid extract of maize kernels non-inoculated or inoculated with AFC-1 (WT) and the 3 deleted Aflox1 strains (1.1/3.2/5.1) at 3, 7, 10 and 14 dpi. The results of the PCA analysis referred to three separate experiments performed in duplicate. [file Presentation2.PPTX]

## Slide 1
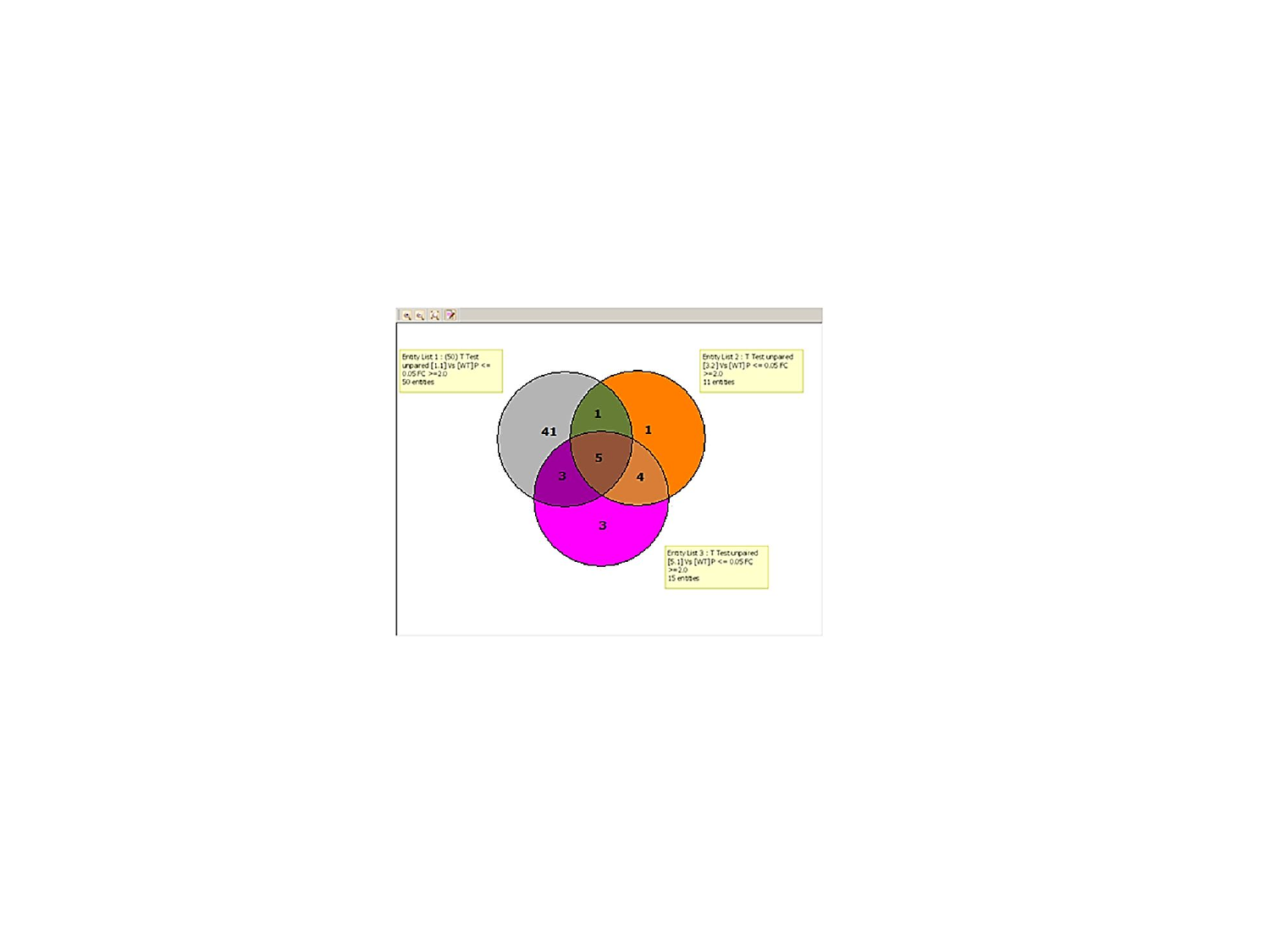

Supplement: Figure S3 — Eulero–Venn's diagram showing compounds differences present in maize kernels inoculated with AFC-1 (WT) or the 3 deleted Aflox1 strains. The labels of each circle report the number of compounds selected on the basis of significant difference and 100% frequency in at least one group. [file Presentation3.PPTX]
